# Characteristics and evolution of pelvic floor structures in female patients aged over 40 years with constipation—a retrospective cohort study

**DOI:** 10.7717/peerj.20783

**Published:** 2026-02-13

**Authors:** Jiarong You, Ying Chen, Rongpu Liang, Li Lu, Jianming Yang, Jiannan Ren, Guangchun Jiang, Yuan Wang, Runze Wu, Xinling Zhang, Bo Wei

**Affiliations:** 1 Department of Gastrointestinal Surgery, Lingnan Hospital, The Third Affiliated Hospital of Sun Yat-sen University, Guangzhou, Guangdong Province, China; 2 Department of Gastrointestinal Surgery, The Third Affiliated Hospital of Sun Yat-sen University, Guangzhou, Guangdong Province, China; 3 Pelvic Floor Medical Center, The Third Affiliated Hospital of Sun Yat-sen University, Guangzhou, Guangdong Province, China; 4 Department of Ultrasound, The Third Affiliated Hospital of Sun Yat-sen University, Guangzhou, Guangdong Province, China; 5 Department of Coloproctology, The Sixth Affiliated Hospital of Sun Yat-sen University, Guangzhou, Guangdong Province, China; 6 Guangdong Provincial Key Laboratory of Colorectal and Pelvic Floor Diseases, The Sixth Affiliated Hospital of Sun Yat-sen University, Guangzhou, Guangdong Province, China

**Keywords:** Constipation, Pelvic floor ultrasound, Pelvic floor structure, Propensity score matching analysis, Retrospective study

## Abstract

**Background:**

Pelvic floor dysfunction (PFD) is a common cause of chronic constipation which can reciprocally exacerbate pelvic floor burden. However, the characteristics and evolution of pelvic floor structures in patients with constipation remain unclear. This study investigates the characteristics and evolution of pelvic floor structures in constipated women aged over 40 years.

**Methods:**

Clinical data were collected from female patients undergoing pelvic floor ultrasound at the Third Affiliated Hospital of Sun Yat-sen University from December 2020 to August 2023. Propensity score matching (PSM) minimized confounders between the constipation (*n* = 247) and non-constipation (*n* = 898) groups. We analyzed intergroup differences in ultrasound data and changes in pelvic floor structure over time among constipated patients.

**Results:**

Significant intergroup differences emerged in uterine prolapse (*P* = *0.042*), rectocele (*P* = *0.022*), levator ani hiatus dilation (*P=0.013*), hiatus area (*P* < *0.01*), the position of the uterus (*P* < *0.01*), and rectal ampulla (*P* = *0.017*) at maximal Valsalva maneuver (VM). Multivariate analysis identified rectocele (*P* = *0.023*) and uterine descent at maximal VM (*P* = *0.026*) as positively associated with constipation occurrence. Multiple ultrasonographic evaluations over two years revealed stable pelvic floor anatomy in non-constipated individuals but identified alterations in 78 constipated patients, including increased vesicocele (*P* = *0.039*), uterine prolapse (*P* = *0.019*), perineal hypermobility (*P* = *0.015*), lower bladder (*P* < *0.001*) and rectal ampulla (*P < 0.01*) positions at maximal VM, greater bladder descent (*P* < *0.01*), and enlarged hiatus area (*P* < *0.01*).

**Conclusion:**

This study demonstrates that rectocele and uterine descent at maximal VM exhibit positive associations with constipation. Over time, further descent of the bladder, uterus, and rectum occurs in female patients with constipation, along with an increase in perineal mobility and levator ani hiatus area.

## Introduction

Chronic constipation, a prevalent gastrointestinal disorder, manifests as difficult defecation, hard stools, and infrequent bowel movements, persisting for over six months and affecting approximately 15% of the global population (Bharucha & Lacy, 2020). Previous studies have shown that chronic constipation is often observed in older women with lower socioeconomic status and comorbid irritable bowel syndrome (IBS), significantly impacting their quality of life (Suares & Ford, 2011).

Chronic constipation is commonly attributed to factors including inadequate dietary fiber intake, sedentary behavior, psychological stress, disorders of colonic propulsion or rectal evacuation, and certain medications. According to the Rome IV criteria, chronic constipation is classified into four subtypes: (a) functional constipation, (b) irritable bowel syndrome (IBS) with constipation, (c) opioid-induced constipation, and (d) functional defecation disorders, encompassing inadequate propulsive force and dyssynergic defecation. Current therapeutic strategies, targeting the underlying causes or triggers, encompass lifestyle and dietary modifications, pharmacotherapy, biofeedback therapy, rectal irrigation/stimulation therapies, and surgical intervention (Aziz et al., 2020; Camilleri et al., 2017). However, a subset of patients experiences suboptimal treatment outcomes, necessitating continuous monitoring to evaluate therapeutic efficacy and guide strategy adjustments. While colonic transit studies and defecography are essential post-therapeutic tools for efficacy assessment, their clinical application is limited by procedural complexity and substantial costs (Bharucha & Lacy, 2020).

While previous research has predominantly investigated the roles of pelvic floor muscle dyssynergia and rectoanal pressure alterations in constipation, comparatively less attention has been directed toward morphological changes in pelvic floor organs. In recent years, elucidating the correlation between pelvic floor dysfunction and constipation has progressively emerged as a key research priority. The pelvis constitutes a critical anatomical structure, providing essential support and protection for visceral organs, and facilitating core functions in reproduction, excretion, and movement. Anatomically, the pelvic floor is compartmentalized: the anterior compartment contains the bladder and urethra; the middle compartment houses the vagina and uterus; and the posterior compartment accommodates the sigmoid colon, rectum, and anal canal. Ultrasound, recognized as a rapid, safe, and cost-effective objective imaging modality, has demonstrated effectiveness in evaluating pelvic floor structures, colonic contents, rectal diameter, and other constipation-related parameters. This capability renders it a comprehensive tool for constipation assessment (Bassotti & Maconi, 2024; Riera & Phatak, 2024). Pelvic organ prolapse, a significant contributor to pelvic floor dysfunction, can be dynamically assessed through pelvic floor ultrasound, which visualizes organ positioning and prolapse severity while quantifying levator ani injuries through measurements such as the levator ani hiatus area (Dietz, 2020; Yoon & Gupta, 2024). This technique visualizes organ positioning and prolapse severity while also enabling quantification of levator ani injuries through metrics like the levator ani hiatus area. The temporal evolution of pelvic floor structure and function in constipated patients represents a complex process. Investigating the pelvic floor structural characteristics in chronic constipation patients *via* ultrasound may thus provide a novel perspective and methodology for monitoring therapeutic outcomes following interventions, potentially circumventing the clinical limitations of traditional assessment methods.

Currently, no systematic investigations have documented the characteristics of pelvic floor structures or their longitudinal evolution in female patients with constipation. This retrospective cohort study therefore aims to characterize pelvic floor structures and their temporal changes in chronically constipated women aged over 40 years. Utilizing pelvic floor ultrasound data from the Third Affiliated Hospital of Sun Yat-sen University, we applied predefined inclusion/exclusion criteria to establish the cohort, performed propensity score matching, and compared structural differences between constipated and non-constipated patients. Serial examinations further enabled analysis of the temporal dynamics of pelvic floor structures in the constipation group. These findings are anticipated to provide novel insights into constipation pathogenesis, elucidate potential progression mechanisms, and deliver evidence-based guidance for clinical diagnosis and management.

## Materials & Methods

### General information

Given the higher prevalence of pelvic floor dysfunction in older women compared to men, we conducted a retrospective analysis of female patients aged over 40 years with a history of constipation who visited the Third Affiliated Hospital of Sun Yat-sen University from December 2020 to August 2023. The ultrasound data were sourced from the Ultrasound Department of the same institution. This study was conducted following the principles of the Declaration of Helsinki and STROBE guidelines and was approved by the Ethics Committee of the Third Affiliated Hospital of Sun Yat-sen University (approval number: [2020] 02-150-01). Written informed consent was incorporated into the questionnaire, and all participants provided their explicit consent, confirming they were fully informed about the study and voluntarily agreed to participate.

### Inclusion and exclusion criteria

This study enrolled 247 female patients with constipation and 898 without. The inclusion criteria for all patients were as follows: (1) Age ≥ 40 years; (2) previous pelvic floor ultrasound examination performed at the Third Affiliated Hospital of Sun Yat-sen University. The exclusion criteria were as follows: (1) Diseases affecting intestinal function (*e.g.*, tumors); (2) current use of medications affecting gastrointestinal motility (*e.g.*, opioids); (3) history of hysterectomy; (4) incomplete clinical data; (5) cognitive or psychiatric disorders that preclude comprehension or accurate completion of questionnaires; (6) declined participation in the study.

### Ultrasound examination method

This study was conducted with the assistance of four-dimensional transperineal pelvic floor ultrasound (TPUS), in which participants were in the lithotomy position with an empty bladder. TPUS offers enhanced visualization of pelvic floor structures and function, providing intuitive, detailed, and dynamic assessment capabilities. To ensure the objectivity and reproducibility of ultrasonographic examination results, all sonographers underwent standardized structured training, with repeated measurements systematically performed during the imaging procedures. Patients with constipation or pelvic floor dysfunction were advised to undergo regular follow-up assessments, typically scheduled at 3- to 6-month intervals. These evaluations included pelvic floor structural and functional examinations to monitor disease progression.

### Questionnaire

Chronic constipation is defined as constipation with symptoms characterized by symptoms persisting for at least 6 months, with at least two of the following criteria met within the preceding 3 months: (1) Straining during more than 25% of bowel movements, (2) more than 25% of bowel movements characterized by lumpy or hard stools, (3) more than 25% of bowel movements associated with a sensation of incomplete evacuation, (4) more than 25% of bowel movements with difficulty, (5) more than 25% of bowel movements requiring manual assistance, (6) spontaneous bowel movements (SBMs) occurring less than three times per week. The diagnosis of functional constipation was assessed by gastroenterologists according to the Rome IV criteria (Mearin et al., 2016). Further, the questionnaire incorporated patient demographic data and constipation-related risk factors, including age, prolonged standing, heavy lifting, chronic cough, and obesity, among others. Additionally, an annual questionnaire survey will be administered to re-evaluate constipation in patients according to the latest Rome diagnostic criteria.

### Outcome measures

The outcome measures comprise differences between patients with constipation and those without constipation, as well as the differences between two pelvic floor ultrasound assessments approximately 2.5 years apart. Specifically, the assessed parameters include: bladder neck mobility, vesicocele, uterine prolapse, rectocele, perineal hypermobility, bladder descent, the position of the bladder, uterus, and rectal ampulla, the area of the levator ani hiatus and whether the hiatus is balloon during maximal Valsalva maneuver (VM), and pelvic organ positions at rest and during maximal VM relative to the inferior margin of the symphysis pubis in the midsagittal plane. The pelvic organs’ descent is defined as the positional difference between maximal VM and rest (Dietz, Shek & Clarke, 2005).

### Statistical methods

Continuous variables were analyzed using the Student’s *t*-test or the Mann-Whitney U test, while categorical variables were assessed with the Chi-square test or Fisher’s exact test. For paired measurements at two time points, mean differences were compared using the paired Student’s *t*-test, and proportional differences were examined with McNemar’s test. The Cox regression model was applied for multivariate analysis, with variables showing a *p*-value <0.10 in the univariate analysis being eligible for inclusion in the Cox regression model.

To mitigate selection bias and potential confounding, Propensity score and nearest neighbor matching (PSM) analysis *via* a nearest-neighbour 1:3 matching scheme with a caliper size of 0.1 was performed using R software. All statistical analyses were performed using GraphPad Prism 9 and R statistical software (version 4.2.2; R Core Team, 2022). R packages, including MatchIt, tableone, and ggplot2, were utilized for statistical analysis and table creation. Statistical significance was defined as *p* < 0.05.

## Results

### Baseline characteristics

**Figure 1:**
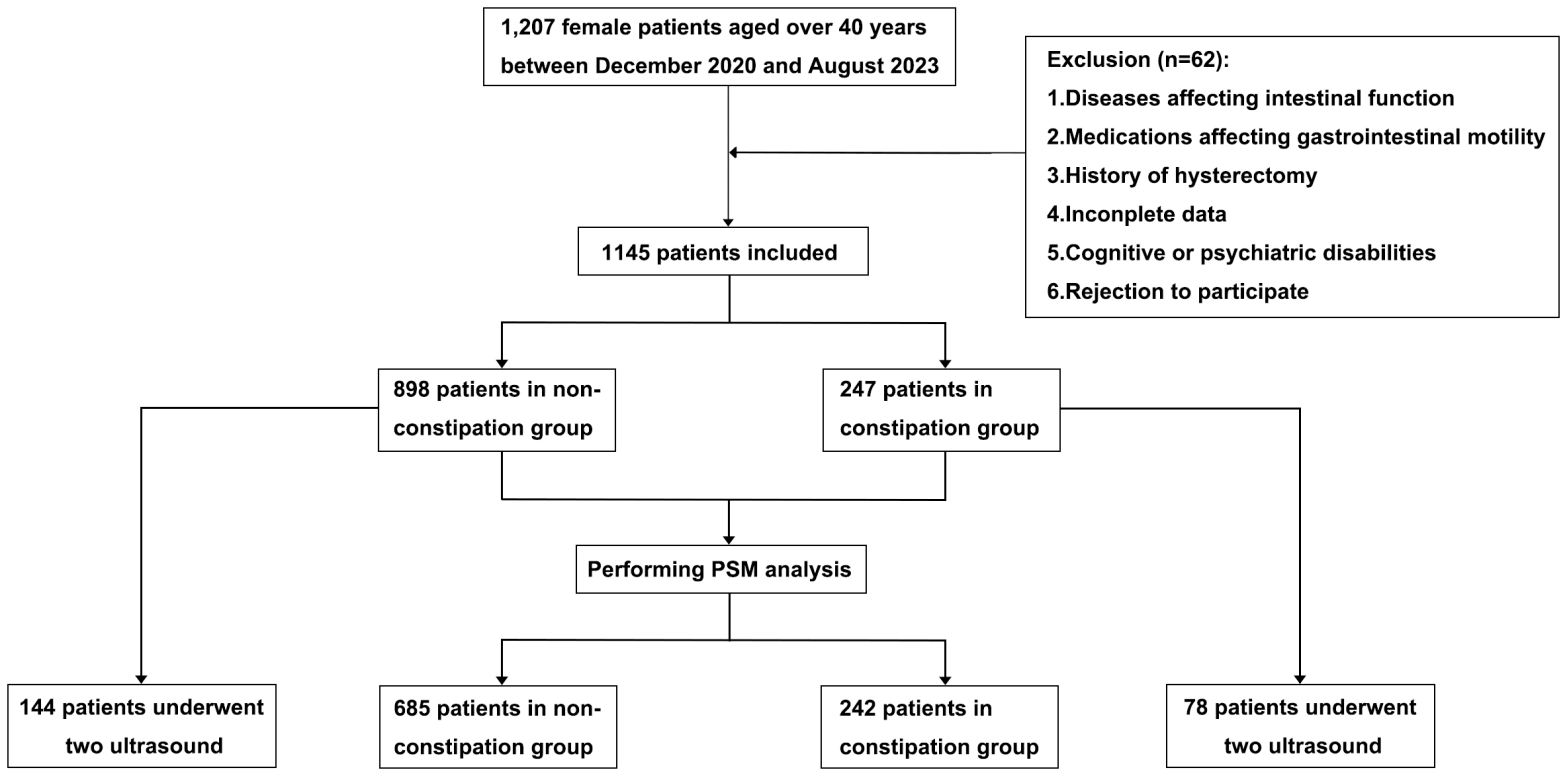
Flowchart. Among 1,207 patients, following the application of the exclusion criteria, a cohort of 1,145 patients was ultimately included in the analysis.

A constipation-related questionnaire survey was administered to female patients attending our hospital from December 2020 to August 2023, followed by a retrospective analysis of their clinical records. Of 1207 patients meeting the selection criteria, 1,145 were ultimately enrolled and analyzed, subsequently stratified into constipation and non-constipation groups. From the entire cohort, 78 constipated and 144 non-constipated patients who had undergone previous pelvic floor ultrasound examinations were identified (Fig. 1).

The baseline characteristics of the constipation group and the non-constipation group are shown in Table 1. Within the entire cohort of 1,145 patients, the constipation group comprised 247 patients, compared to 898 in the non-constipation group. There were no significant differences in most variables between the two groups, except for the median age of the constipation group being greater than that of the non-constipation group (Median, 51 y *vs.* 49 y, *P* = 0.006). Following propensity score matching in a 1:3 ratio, 242 patients from the constipation group were matched to 685 patients from the non-constipation group; baseline characteristics were balanced, with no significant group differences observed.

**Table 1:** Baseline characteristics of the constipation group and non-constipation group in the entire cohort and PSM cohort.

	Entire cohort (*n* = 1, 145)			PSM cohort (*n* = 927)		
Characteristic	Non-constipation (*n* = 898)	Constipation (*n* = 247)	*P*	Non-constipation (*n* = 685)	Constipation (*n* = 242)	*P*
Long standing and lifting (%)			0.789			0.926
yes	169(18.8)	44(17.8)		118(17.2)	43(17.8)	
no	729(81.2)	203(82.2)		567(82.8)	199(82.2)	
Macrosomia delivery (%)			0.836			0.797
yes	11 (1.2)	2 (0.8)		9 (1.3)	2 (0.8)	
no	887 (98.8)	245 (99.2)		676 (98.7)	240 (99.2)	
Age (y, median (IQR))	49.0 [42.0, 57.8]	51.0 [43.0, 62.0]	0.006	50.0 [43.0, 59.0]	51.0 [43.0, 61.8]	0.301
Chronic cough (%)			0.602			0.867
yes	38 (4.2)	13 (5.3)		32 (4.7)	10 (4.1)	
no	860 (95.8)	234 (94.7)		653 (95.3)	232 (95.9)	
Obesity (%)			1			0.934
yes	31 (3.5)	9 (3.6)		20 (2.9)	8 (3.3)	
no	867 (96.5)	238 (96.4)		665 (97.1)	234 (96.7)	

### Pelvic floor ultrasound for multifaceted assessment of pelvic floor function

**Figure 2:**
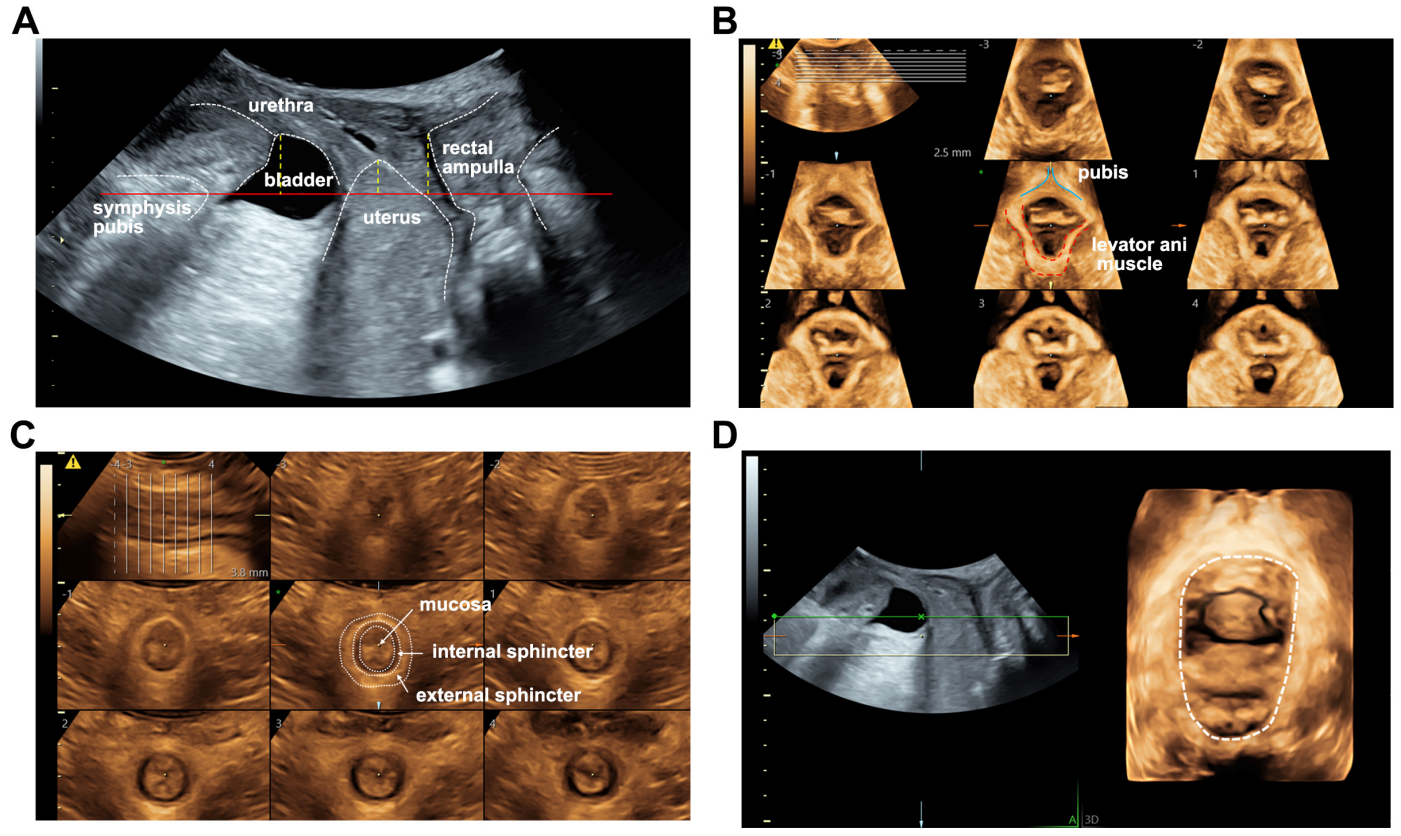
Pelvic floor ultrasound images of a 47-year-old woman with constipation. (A) Mid-sagittal gray-scale image indicated the vesicocele and uterine prolapse. The horizontal line (the red solid line) was the reference line passing through the posterior inferior margin of the symphysis pubis. The vertical line (the yellow dotted line) indicated the distance from the bladder, uterus, and rectal ampulla to the reference line. (B) Tomography ultrasound images showed the left side levator ani muscle was trauma. (C) Tomography ultrasound images of the anal sphincter was normal. (D) The levator hiatus area (the white line) at the maximal Valsalva maneuver was over 20 cm^2^.

As described in Fig. 2, the reference line is the horizontal line at the posterior inferior margin of the pubic symphysis. Normal bladder neck descent measures <25 mm; descent ≥25 mm indicates bladder neck hypermobility. Vesicocele involves protrusion of the bladder into the vagina, typically affecting the anterior bladder wall in its lower two-thirds. Uterine prolapse is defined as the descent of the uterus from its normal position along the vaginal canal, with the cervical external os descending below the level of the ischial spines, potentially resulting in complete uterine prolapse outside the vaginal opening. Rectocele is characterized by ≥6 mm herniation of the anterior rectal wall into the posterior vaginal wall. Perineal hypermobility denotes descent of the perineal body tissue ≥15 mm vertically from the reference line due to fascial defects. As described in previous paper (International Urogynecology, 2019), an avulsion of levator ani muscle is diagnosed when at least the three central slices on tomography ultrasound image (TUI) show an abnormal insertion of the muscle on the inferior pubic ramus. Anal sphincter injury was defined the 3rd to the 8th slices on the TUI showed a defect of ≥30 degrees of the anal sphincter (Scheer, Thakar & Sultan, 2009). The levator hiatus area larger than 20 cm^2^ was the diagnostic criterion of ballooning (Fig. 2) (Dou et al., 2018; Xiao et al., 2019).

### Logistic regression analysis of pelvic floor ultrasound results in the entire cohort and propensity-matched cohort

Prior to propensity score matching analysis, univariate logistic regression of the entire cohort revealed statistically significant differences in rectocele, levator ani hiatus, and uterine position relative to the pubic symphysis during maximal Valsalva maneuver between the two groups (*P* = 0.020, *P* = 0.048, *P* = 0.033). Multivariate logistic regression analysis suggested that the differences in rectocele were statistically significant (OR, 1.82; 95% CI [1.07–3.02]; *P* = 0.023; Table 2), indicating that the greater the rectocele, the higher the probability of constipation occurrence.

**Table 2:** Univariate and multivariate analyses for constipation occurrence in the entire cohort.

**Characteristics**		Non-constipation (*n* = 898)	Constipation (*n* = 247)	**Univariate**		**Multivariate**	
				OR (95% CI)	*P*	OR (95% CI)	*P*
Bladder neck hypermobility (%)	No	282 (31.4)	78 (31.6)	Reference			
	Yes	616 (68.6)	169 (68.4)	0.99 (0.73–1.34)	0.958		
Vesicocele (%)	No	337 (37.5)	91 (36.8)	Reference			
	Yes	561 (62.5)	156 (63.2)	1 (0.77–1.38)	0.844		
Uterine prolapse (%)	No	580 (64.6)	154 (62.3)	Reference			
	Yes	318 (35.4)	93 (37.7)	1.1 (0.82–1.47)	0.516		
Rectocele (%)	No	848 (94.4)	223 (90.3)	Reference			
	Yes	50 (5.6)	24 (9.7)	1.8 (1.1–3.04)	0.020	1.82 (1.07–3.01)	0.023
Perineal hypermobility (%)	No	537 (59.8)	153 (61.9)	Reference			
	Yes	361 (40.2)	94 (38.1)	0.91 (0.68–1.22)	0.542		
Rupture of the levator ani muscle (%)	No	878 (97.8)	240 (97.2)	Reference			
	Yes	20 (2.2)	7 (2.8)	1.3 (0.54–3.06)	0.579		
Rupture of the anal sphincter (%)	No	892 (99.3)	244 (98.8)	Reference			
	Yes	6 (0.7)	3 (1.2)	1.8 (0.45–7.36)	0.396		
Levator ani hiatus dilation (%)	No	290 (32.3)	67 (27.1)	Reference			
	Yes	608 (67.7)	180 (72.9)	1.3 (0.94–1.75)	0.121		
Levator ani hiatus area (cm^2^, median (IQR))		22.75 [18, 28]	24 [19.85, 29]	1 (1–1.04)	0.048	1.01 (0.99–1.03)	0.449
Position of the bladder at rest (mm, median (IQR))		29 [27, 31]	29 [27, 31]	1 (0.96–1.04)	0.869		
Position of the bladder at VM (mm, median (IQR))		−3 [−10, 9]	−2 [−9.5, 10]	1 (0.99–1.01)	0.569		
Position of the uterus at VM (mm, median (IQR))		6 [−2, 14]	5 [−1, 9]	0.99 (0.98–0.999)	0.033	0.99 (0.98–1.00)	0.154
Position of the rectum at VM (%)	Up	122 (13.6)	23 (9.3)	Reference			
	Down	776 (86.4)	224 (90.7)	1.5 (0.96–2.45)	0.075	1.31 (0.81–2.17)	0.285
Bladder descent (mm, median [IQR])		32 [22, 40]	31 [21, 40]	1 (0.99–1.01)	0.599		

**Notes.**

OR odds ratio VM maximal Valsalva maneuver up/down the position of the pelvic organs is either above or below the pubic symphysis.

Following PSM implementation, univariate regression identified statistically significant differences in uterine prolapse, rectocele, ballooning of levator ani hiatus, hiatus area, and positional variations of both the uterus and rectal ampulla relative to the pubic symphysis at maximal VM (*P* < 0.05). In multivariate analysis, rectocele exhibited a positive correlation with constipation occurrence (OR, 1.90; 95% CI [1.08–3.29]; *P* = 0.023; Table 3), whereas uterine position during maximal VM showed a negative correlation (OR, 0.98; 95% CI [0.97–1.00]; *P* = 0.026). In the constipation group, the proportion of patients with rectocele was 9.5%, significantly higher than the 5.3% observed in the non-constipation group. Furthermore, the uterine position at maximal VM was significantly lower in constipation cases than in controls (median, five mm *vs.* seven mm, *P* < 0.05). Collectively, these findings indicate that rectocele and uterine descent at maximal VM exhibit positive associations with a higher risk of constipation occurrence.

**Table 3:** Univariate and multivariate analyses for constipation occurrence in the PSM cohort.

**Characteristics**		Non-constipation (*n* = 685)	**Constipation (*n* = 242)**	**Univariate**		**Multivariate**	
				OR (95% CI)	*P*	OR (95% CI)	*P*
Bladder neck hypermobility (%)	No	243 (35.5)	74 (30.6)	Reference			
	Yes	442 (64.5)	168 (69.4)	1.2 (0.91–1.71)	0.168		
Vesicocele (%)	No	279 (40.7)	88 (36.4)	Reference			
	Yes	406 (59.3)	154 (63.6)	1.2 (0.89–1.63)	0.233		
Uterine prolapse (%)	No	471 (68.8)	149 (61.6)	Reference			
	Yes	214 (31.2)	93 (38.4)	1.4 (1–1.86)	0.042	0.82 (0.52–1.29)	0.391
Rectocele (%)	No	649 (94.7)	219 (90.5)	Reference			
	Yes	36 (5.3)	23 (9.5)	1.9 (1.1–3.27)	0.022	1.90 (1.08–3.29)	0.023
Perineal hypermobility (%)	No	446 (65.1)	148 (61.2)	Reference			
	Yes	239 (34.9)	94 (38.8)	1.2 (0.88–1.6)	0.271		
Rupture of the levator ani muscle (%)	No	669 (97.7)	235 (97.1)	Reference			
	Yes	16 (2.3)	7 (2.9)	1.2 (0.51–3.07)	0.633		
Rupture of the anal sphincter (%)	No	680 (99.3)	239 (98.8)	Reference			
	Yes	5 (0.7)	3 (1.2)	1.7 (0.4–7.2)	0.466		
Levator ani hiatus dilation (%)	No	238 (34.7)	63 (26.0)	Reference			
	Yes	447 (65.3)	179 (74.0)	1.5 (1.1–2.1)	0.013	1.07 (0.69–1.68)	0.757
Levator ani hiatus area (cm^2^, median (IQR))		22 [18, 28]	24 [20, 29]	1 (1–1.05)	0.003	1.01 (0.98–1.04)	0.392
Position of the bladder at rest (mm, median (IQR))		29 [27, 31]	29 [27,31]	1 (0.97–1.06)	0.559		
Position of the bladder at VM (mm, median (IQR))		−2 [−10, 10]	−2 [−10, 9]	0.99 (0.98–1.01)	0.358		
Position of the uterus at VM (mm, median (IQR))		7 [−1, 14]	5 [−2,9]	0.98 (0.97–0.993)	0.001	0.98 (0.97–1.00)	0.026
Position of the rectum at VM (%)	Up	105 (15.3)	22 (9.1)	Reference			
	Down	580 (84.7)	220 (90.9)	1.8 (1.1–2.94)	0.017	1.46 (0.89–2.48)	0.150
Bladder descent (mm, median (IQR))		31 [21, 39]	31 [21.7, 40]	1 (0.99–1.02)	0.278		

**Notes.**

OR odds ratio VM maximal Valsalva manoeuvre up/down the position of the pelvic organs is either above or below the pubic symphysis.

### Comparative analysis of pelvic floor structure in constipation patients between the two examinations

**Figure 3:**
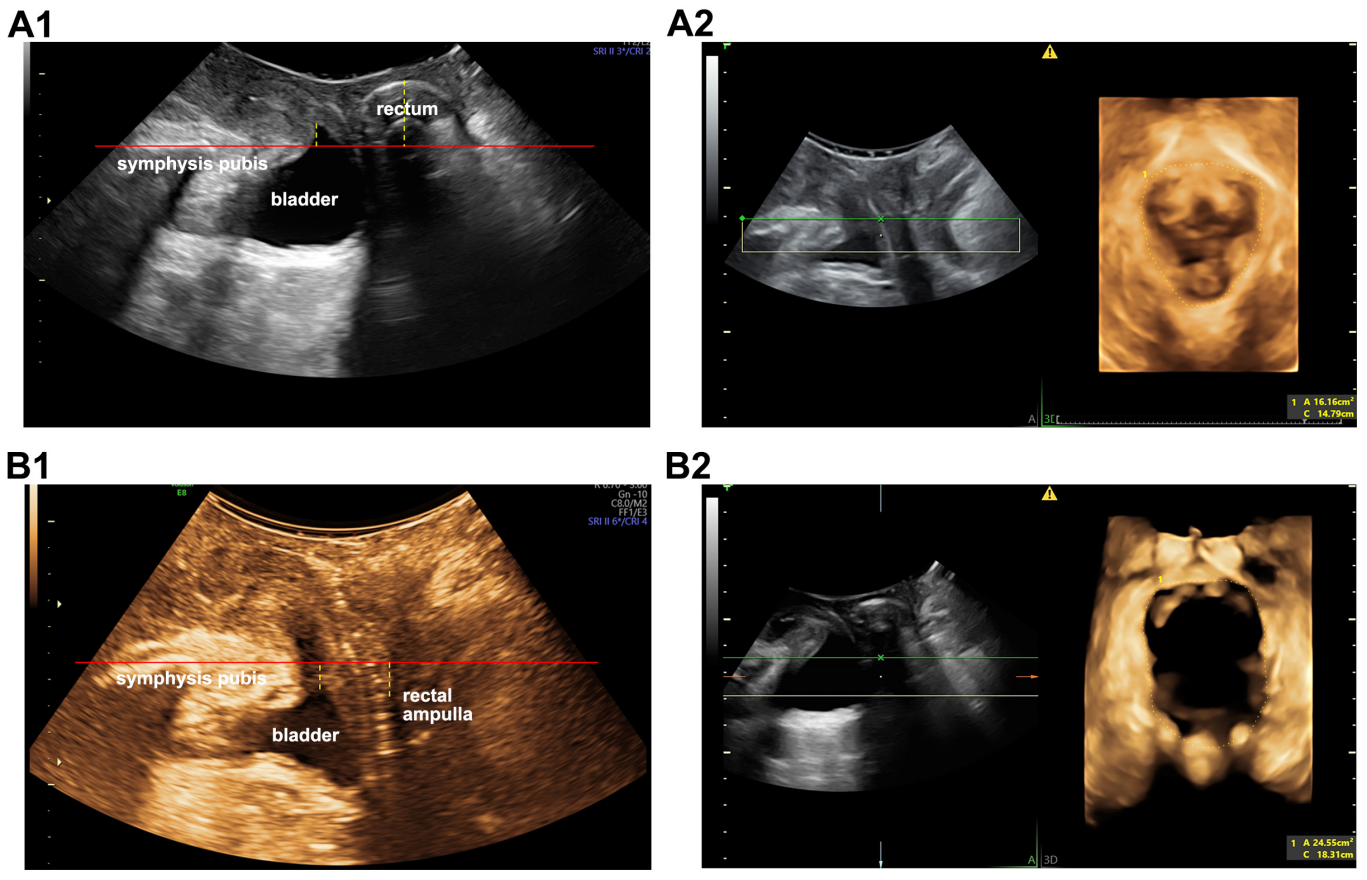
Ultrasound images of the patient with constipation before and after 7 years. (A1) Images in 2015 were without any positive features. (A2) The levator hiatus area in 2015 year was 16.16 cm^2^. (B1) Images in the 2022 year were diagnosed as vesicocele and rectocele. (B2) The levator hiatus area was 24.55 cm^2^.

Interestingly, 78 patients in the constipation group and 144 in the non-constipation group had previously undergone ultrasound examinations. Consequently, the pelvic floor structural changes between the two ultrasound assessments in these patients were analyzed further. Among non-constipated patients, no significant changes in pelvic floor anatomy were detected over a median interval of 2.25 years [IQR, [1.25,3.29]; Fig. S1]. In contrast, constipation patients exhibited structural modifications over time. As shown in Fig. 3, we identified a patient with constipation who, upon a follow-up ultrasound examination after 7 years, developed vesicocele, rectocele, and ballooning of levator ani hiatus.

**Figure 4:**
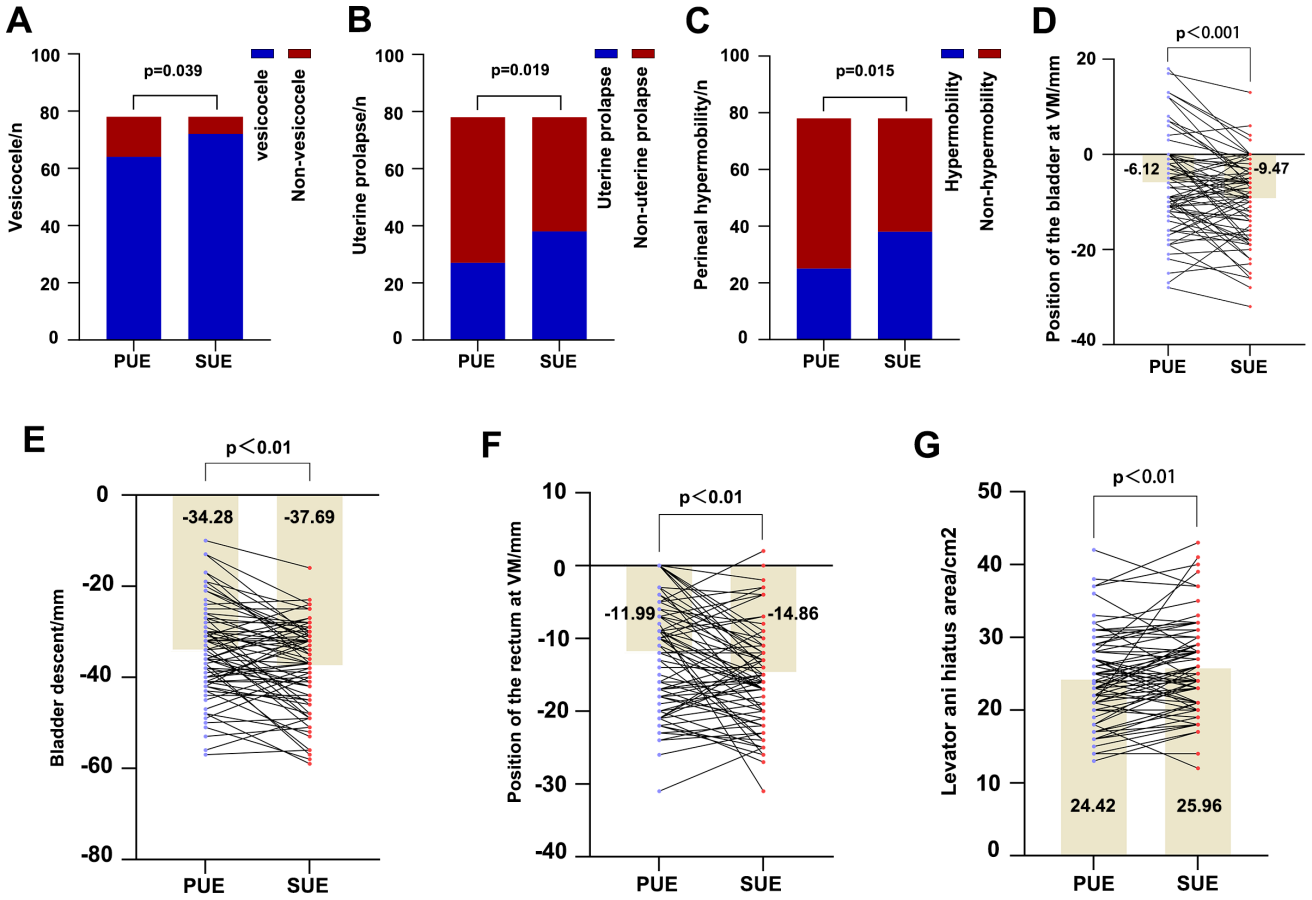
The positive results of the comparative analysis between the two examinations in patients with constipation. Comparison of pelvic floor structures observed in patients with constipation between two exams. The unit on the *y*-axis in plots (A–C) is “n”, which denotes the number of participants. Tan colored bars indicate group means, labeled with values; dots and lines represent paired measurements per patient in (D–G). PUE: Previous Ultrasound Examination; SUE: Subsequent Ultrasound Examination.

The median interval between the two TPUS examinations for these constipation patients was 2.5 years (IQR, [1.40,3.77]). Comparative results between the two examinations are detailed in Fig. 4 and Fig. S2. No significant differences emerged in bladder neck mobility, rectocele, ballooning of levator ani hiatus, levator ani muscle avulsion or anal sphincter injury, or the bladder position at rest. In the subsequent examination, the position of the bladder and rectal ampulla relative to the symphysis pubis at maximal VM was significantly lower, and both the bladder descent and levator ani hiatus area were larger compared to the previous examination. Moreover, a higher proportion of patients exhibited vesicocele, uterine prolapse, and perineal hypermobility at the subsequent examination.

## Discussion

Chronic constipation is a prevalent condition that adversely affects quality of life and social productivity; it is also associated with severe long-term outcomes, including malnutrition, coronary heart disease, and ischemic stroke (Sumida et al., 2019; Wang et al., 2024). Constipation symptoms are typically nonspecific and exhibit considerable overlap across various subtypes. In cases where first-line treatments prove ineffective, objective examinations become essential for identifying distinct subtypes and their risk factors, thereby optimizing constipation management (Camilleri et al., 2017). The gastrointestinal symptom questionnaire serves as an efficient tool for symptom assessment (Bharucha & Lacy, 2020). This study utilized a questionnaire format to quickly screen constipation patients for further research. Pelvic floor dysfunction (PFD) represents a major factor contributing to constipation, with defecography constituting the first-line diagnostic method for refractory cases (Grossi et al., 2018). Defecography evaluates the functional and anatomical aspects of the anorectum, whereas magnetic resonance (MR) defecography provides more comprehensive pelvic floor anatomical details (Carter et al., 2020; Salvador et al., 2019). However, MR defecography is performed in a non-physiological supine position and limited by high costs and long examination times. Conversely, ultrasound examination offers a common accessible imaging modality for evaluating pelvic floor anatomy and dysfunction (Haylen et al., 2010); it additionally assesses fecal retention and gas distribution, reflecting chronic constipation patients’ responses to pharmacological interventions (Manabe et al., 2018; Manabe et al., 2019). Ultrasound enables comprehensive constipation evaluation, and recent studies indicate its potential in providing objective evidence for early diagnosis, prevention, intervention, and therapeutic efficacy assessment in pelvic floor dysfunction (Yao et al., 2021). For women presenting with posterior pelvic symptoms, ultrasound represents the preferred imaging modality (Dietz & Beer-Gabel, 2012).

The pelvic floor anatomy is complex and intricate, consisting of multiple layers of muscles and fascia that close the pelvic outlet and support pelvic organs. This structure can be divided into three layers: the pelvic diaphragm (innermost layer), the urogenital diaphragm (middle layer), and the superficial perineal fascia and superficial muscle layer (outer layer), traversed anteroposteriorly by the urethra, vagina, and rectum. PFD refers to a spectrum of prevalent symptoms and anatomical changes stemming from abnormal pelvic floor muscle function. These functional disorders involve hyper- or hypoactivity or poor coordination of the pelvic musculature (Grimes & Stratton, 2024). Constipation resulting from pelvic floor dysfunction or functional defecation disorders represents one of common pelvic floor disorders in women. Therefore, investigating the characteristics and evolution of pelvic floor structures in constipated patients may facilitate a better understanding of the condition’s pathogenesis and progression, potentially aiding the standardization of its diagnosis and management.

Rectocele predominantly affects the posterior compartment, with an incidence of 30–50% in women over 50 years old. Common symptoms include difficulty in defecation, the need for digital evacuation, pelvic heaviness, anal incontinence, or pain (Aubert et al., 2021; Drutz & Alarab, 2006). Although defecography remains the diagnostic gold standard, studies suggest comparable accuracy of ultrasound when applying identical evaluation criteria (Beer-Gabel & Carter, 2015; Hainsworth et al., 2017). Notably, this study demonstrates a significant positional difference in the rectal ampulla relative to the pubic symphysis at maximal VM between constipated and non-constipated cohorts (*P* = 0.017). Rectocele demonstrates a significant positive association with constipation presence, suggesting that patients with this anatomical defect face elevated risks of developing constipation. Related studies have shown that the size of rectocele is positively correlated with heightened pre-defecation anal sphincter pressure (Sun et al., 2023), and anal manometry has been proposed to support chronic constipation diagnosis (Bharucha & Rao, 2014; Camilleri et al., 2017). Rectoceles are highly prevalent yet frequently asymptomatic. Among symptomatic individuals, reported associations between defecatory difficulty and rectocele vary substantially across studies. Determining the extent to which rectocele contributes to constipation symptoms requires further prospective research for validation (Tirumanisetty et al., 2018). Furthermore, this study found no statistically significant intergroup difference in perineal hypermobility—a finding congruent with Dietz et al.’s conclusion that perineal hypermobility is not an independent predictor of obstructed defecation (Dietz et al., 2021).

Accumulating evidence identifies uterine prolapse as a significant risk factor for functional constipation (Ng et al., 2002). Emerging evidence demonstrates that in women with deep infiltrating endometriosis, pelvic floor hypertonicity, such as levator ani muscle coactivation, exhibits a strong correlation with chronic constipation (Del Forno et al., 2021; Raimondo et al., 2022). A lower uterine position at maximal VM may indicate insufficient pelvic floor relaxation, which impedes intra-abdominal pressure transmission, thereby elevating constipation risk (Youssef et al., 2020; Youssef et al., 2021). Anatomically, the uterus lies anterior to the rectum; severe prolapse or retroversion may directly compress rectal structures or disrupt puborectalis muscle function in effectively forming the anorectal angle, leading to constipation. Moreover, chronic defecation difficulties may induce pelvic neuropathic injury, potentially impairing overall pelvic floor function and exacerbating uterine prolapse. In this study, a significantly higher proportion of uterine prolapse was observed in the constipation group than in the non-constipation group (38.4% *vs.* 31.2%, *P* = 0.042). Multivariate analysis confirmed an inverse relationship between uterine position relative to the pubic symphysis at maximal VM and constipation occurrence. Consequently, uterine preservation with appropriate positional elevation during prolapse management may mitigate pelvic floor dysfunction and constipation risk. Monotherapy may inadequately address constipation, especially in cases with concurrent pelvic floor dysfunction, underscoring the necessity for post-intervention ultrasound evaluation of pelvic anatomy to optimize therapeutic outcomes.

Constipation, a risk factor for non-monocausal enuresis in children (Rodríguez-Ruiz et al., 2021), significantly increases the likelihood of urinary system dysfunction compared to individuals with normal bowel function (Axelgaard et al., 2023; Iguchi et al., 2021; Sampaio et al., 2016; White et al., 2022). Additionally, studies indicate a high prevalence of constipation among middle-aged women experiencing urinary dysfunction (Averbeck & Madersbacher, 2011), potentially attributable to the close anatomical proximity and shared neural innervation of the two systems. However, some literature has reported no significant impact of chronic constipation on bladder storage or emptying functions (Naseri et al., 2022). In this study, no significant differences were observed between the constipation and non-constipation groups regarding bladder neck mobility, cystocele, or bladder position relative to the pubic symphysis either at rest or during maximal VM (*P* = 0.168, *P* = 0.233). However, worsening constipation symptoms correlated with a marked descent of the bladder position during maximal VM and a significant increase in the incidence of vesicocele (*P* < 0.001, *P* = 0.019).

The levator ani complex constitutes the core support structure of the pelvic floor. Compromise of its function may lead to pelvic organ prolapse and related pelvic floor disorders. The levator ani hiatus area provides a direct measure of the hiatus size, reflecting the biomechanical characteristics of the levator ani to some extent (Dietz, 2021). Consistent with this, our results confirm that patients with chronic constipation exhibit significant levator ani hiatal ballooning and an increased hiatal area compared to non-constipated individuals (*P* = 0.013, *P* < 0.01). This study also revealed that only a minority of patients presented with levator ani muscle avulsion or anal sphincter injury; such injury appears to be more strongly associated with fecal incontinence (Heilbrun et al., 2010).

Pelvic floor anatomical dysfunction contributes to constipation onset, while constipation can further exacerbate pelvic floor structural and functional damage. However, there is currently no specific literature reporting on the changes in pelvic floor structures of patients with constipation over time. Utilizing TPUS examinations, we evaluated pelvic floor structural changes over an approximately 2.5-year period in patients with and without constipation. Our findings indicate stability in pelvic floor structures among non-constipated individuals over a median 2.25-year period. Conversely, constipated patients exhibited a significant descent of the bladder and rectal ampulla relative to the pubic symphysis at maximal VM over time. Additionally, the difference in bladder position between rest and maximal VM increased, accompanied by levator ani hiatal enlargement. Constipated patients also demonstrated higher prevalence rates of vesicocele, uterine prolapse, and perineal hypermobility. These longitudinal observations indicate progressive pelvic floor structural deterioration in constipation, likely attributable to chronic stimuli such as excessive defecatory straining, which damages support structures (Chamié et al., 2018; Kobi et al., 2018). Early clinical intervention is therefore critical to mitigate this progression.

Several limitations warrant acknowledgment. First, the etiology of constipation is multifactorial, and imaging alone cannot account for all cases. Hence, we still need to combine additional diagnostic tests, such as colon transit studies, anorectal manometry, and sensory function assessments, to evaluate constipation comprehensively. Second, the study cohort was exclusively recruited from female patients aged over 40 years, inherently restricting the generalizability of these findings to younger populations and male patients. Third, the relatively short observation period may be insufficient to characterize long-term structural evolution within the pelvic floor among constipated individuals, highlighting the necessity for extended longitudinal investigations. Fourth, as a single-center, observational, retrospective study, the sample size may be limited with potential selection bias.

## Conclusions

This study reveals a positive association between rectocele, uterine descent at maximal VM, and constipation occurrence, indicating that pelvic floor structural abnormalities constitute a pivotal pathophysiological mechanism underlying chronic constipation and may represent critical imaging biomarkers for constipation risk assessment and post-therapeutic monitoring. Moreover, longitudinal observation revealed a certain degree of pelvic floor structural deterioration and descent within the constipated patients, manifested by higher incidences of vesicocele, uterine prolapse, and perineal hypermobility over time. Quantitatively confirmed were lower positions of the bladder and rectal ampulla relative to the pubic symphysis at maximal VM, coupled with greater bladder mobility and an enlarged levator ani hiatus area longitudinally. Critically, these temporal structural changes significantly advance current understanding of constipation pathogenesis and provide novel mechanistic perspectives for investigating its development. We still need to conduct larger-scale multicenter clinical trials to validate these results.

## Supplemental Information

10.7717/peerj.20783/supp-1 Supplemental Information 1 The Comparative Analysis between the two examinations in patients without constipation Comparison of pelvic floor structures observed in patients without constipation between two exams. The unit on the y-axis in plots (A–H) is “n”, which denotes the number of participants. Tan colored bars indicate group means, labeled with values; dots and lines represent paired measurements per patient in (I–M). PUE: Previous Ultrasound Examination; SUE: Subsequent Ultrasound Examination.

10.7717/peerj.20783/supp-2 Supplemental Information 2 The negative results of the Comparative Analysis between the two examinations in patients with constipation Comparison of pelvic floor structures observed in patients with constipation between two exams. The unit on the y-axis in plots (A–E) is “n”, which denotes the number of participants. Tan colored bars indicate group means, labeled with values; dots and lines represent paired measurements per patient in (F). PUE: Previous Ultrasound Examination; SUE: Subsequent Ultrasound Examination.

10.7717/peerj.20783/supp-3 Supplemental Information 3 Raw data 2. two ultrasounds This dataset includes patients who underwent two ultrasound examinations, comprising 78 patients with constipation and 144 patients without constipation, all included in the analysis.

10.7717/peerj.20783/supp-4 Supplemental Information 4 Raw data 1 This dataset includes 1,145 patients with constipation and non-constipation cases.

10.7717/peerj.20783/supp-5 Supplemental Information 5 Questionnaire The questionnaire includes the patient’s informed consent regarding the purpose of the questionnaire and the content of the questionnaire.

10.7717/peerj.20783/supp-6 Supplemental Information 6 STROBE-checklist

## Additional Information and Declarations

### Data Availability

The following information was supplied regarding data availability: The raw data is available in the Supplemental Files.
