# Supplementary figures and images for: Characteristics and evolution of pelvic floor structures in female patients aged over 40 years with constipation—a retrospective cohort study
Source: PeerJ. 2026 Feb 13;14:e20783. doi: 10.7717/peerj.20783 (PMC12908577; doi:10.7717/peerj.20783)

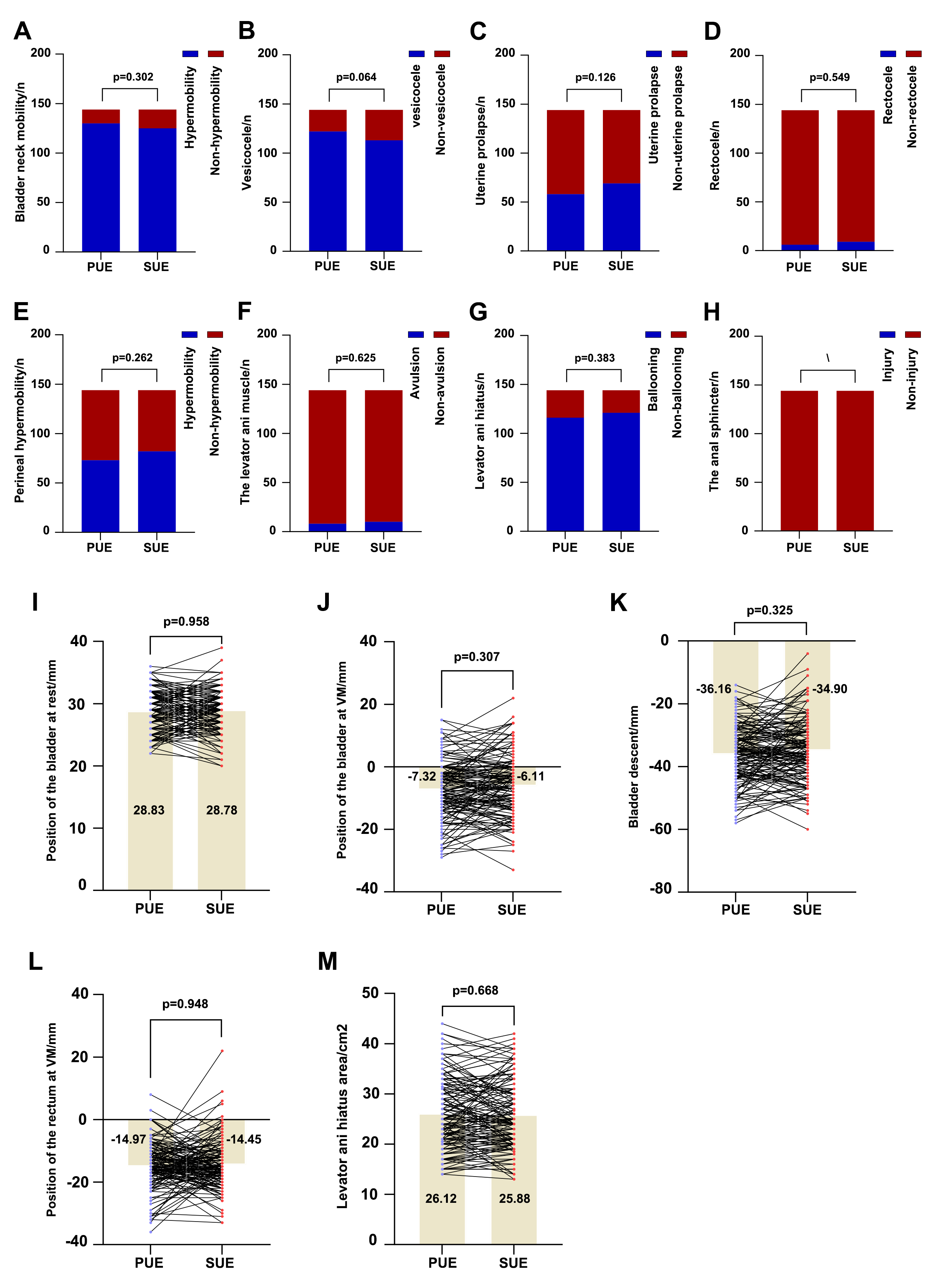

Supplement: Supplemental Information 1 — Comparison of pelvic floor structures observed in patients without constipation between two exams. The unit on the y-axis in plots (A–H) is “n”, which denotes the number of participants. Tan colored bars indicate group means, labeled with values; dots and lines represent paired measurements per patient in (I–M). PUE: Previous Ultrasound Examination; SUE: Subsequent Ultrasound Examination. [file peerj-14-20783-s001.png]

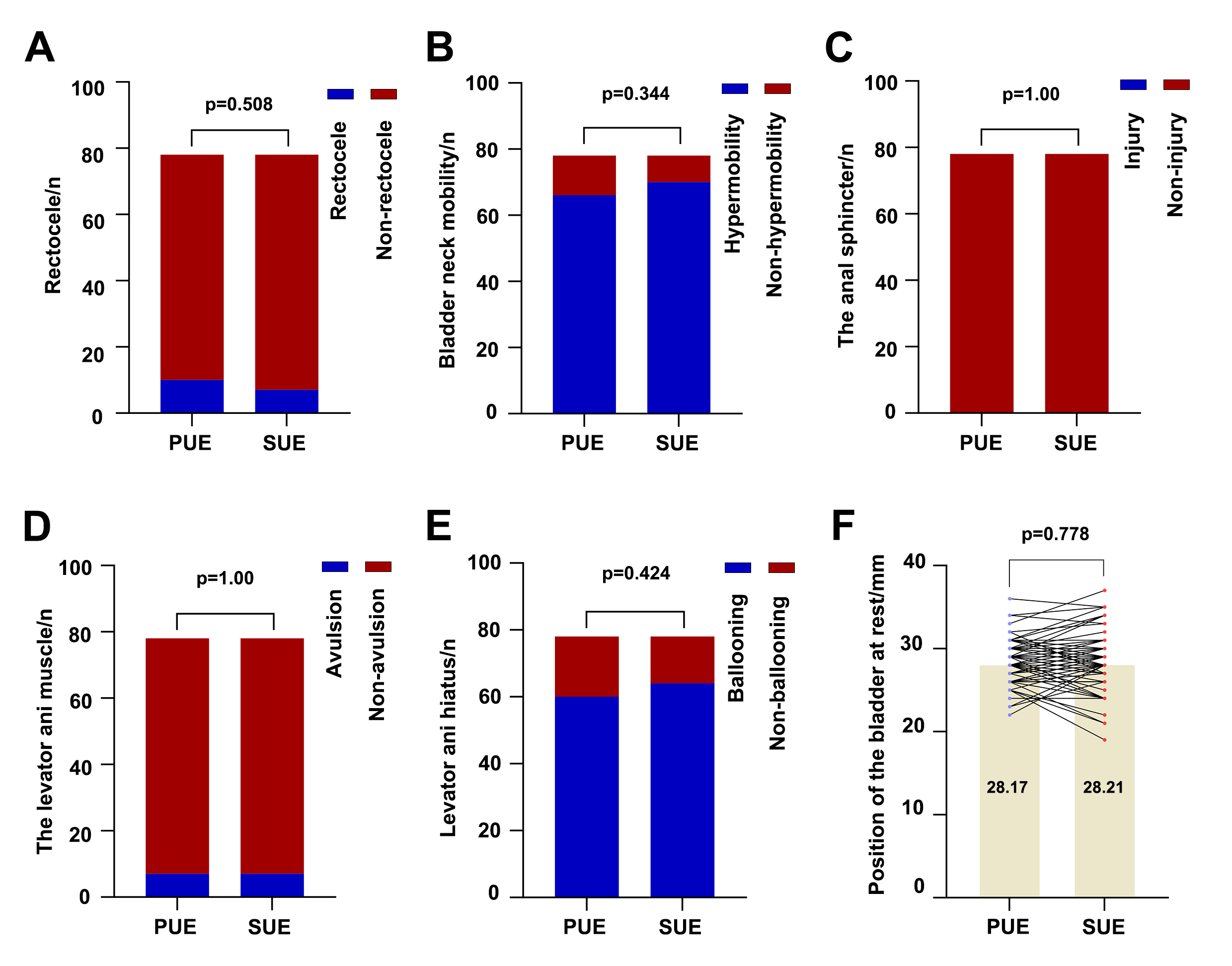

Supplement: Supplemental Information 2 — Comparison of pelvic floor structures observed in patients with constipation between two exams. The unit on the y-axis in plots (A–E) is “n”, which denotes the number of participants. Tan colored bars indicate group means, labeled with values; dots and lines represent paired measurements per patient in (F). PUE: Previous Ultrasound Examination; SUE: Subsequent Ultrasound Examination. [file peerj-14-20783-s002.png]
