# Supplementary material for: Characteristics and evolution of pelvic floor structures in female patients aged over 40 years with constipation—a retrospective cohort study
Source: PeerJ. 2026 Feb 13;14:e20783. doi: 10.7717/peerj.20783 (PMC12908577; doi:10.7717/peerj.20783)
Supplement: Supplemental Information 5 — The questionnaire includes the patient’s informed consent regarding the purpose of the questionnaire and the content of the questionnaire. [file peerj-14-20783-s005.docx]

**问 卷 调 查 知 情 同 意 书**

**研究标题：**盆底超声在慢性便秘评估和预测中的价值。

**项目负责人及单位：**魏波、张新玲，中山大学附属第三医院胃肠外科、超声科

尊敬的参与者：

目前正在进行一项关于使用超声来探索便秘患者盆底结构特征的研究，这将有助于更深刻地理解便秘发生的盆底解剖，也可能借此完善便秘的诊疗规范。我们邀请您参加一项问卷调查。参与这项问卷调查完全是自愿的，如您不想参加，您的医疗待遇与权益不会受到影响。您也可以随时改变主意并退出问卷调查。本项目已通过医学伦理委员会审查通过。

**参与本项目的条件**

参与本项问卷调查，您必须满足以下条件：

- 年满40岁或以上
- 曾在中山大学附属第三医院接受过盆底超声检查
- 具备理解问卷和正确回答的能力

**为何要进行本项目？**

本项目的是探索盆底结构改变对女性患者便秘的影响。

**本项目如何进行？**

这项问卷调查将询问有关您 便秘与否及相关危险因素 的问题，题目类型为 选择题和填空题 。调查问卷主要通过微信、问卷星等网络平台或由研究人员在诊室、病房进行发放。如您同意参加，您需要完成问卷填写，我们将收集您所填内容进行统计分析。

**需花费的时间/费用？**

问卷将花费您大约 5 分钟。参加本项目不会增加您的费用。

**风险与赔偿/个人隐私保护和数据安全**

参与本项目不会影响您的诊疗，不增加风险。

如发生与本项目相关的损害，经国家法律法规规定的权威机构认定需要承担相应责任的，按照国家法律法规进行赔偿。

我们将会收集您的个人信息： 姓名，年龄，身高，体重。

您的个人隐私会得到保护，我们会对您所提供的数据进行保密。您的数据将会被储存于 加密的硬盘 ，并保存直至研究结束。仅本项目研究人员可以查阅；必要时，政府管理部门、研究单位监管部门或伦理审查委员会可按规定查阅。

我们可能会将本项目的发现和结果用于文章发表，但您的可识别身份信息不会被公布。

您的个人信息和问卷填写内容我们可能会将去识别化的数据用于未来科学研究：□同意 □不同意。

**联系方式**

如您有任何疑问或需要进一步的信息，请随时与我们联系。研究人员联系方式： 。

如您对于作为研究参与者所享有的权益有任何疑问，请联系：中山大学附属第三医院医学伦理委员会。

**同意声明**

您同意参与此项问卷调查，且满足参与的条件。

您理解参与项目是自愿的，且可以随时撤回知情同意书并退出。

**如您同意上述声明，请点击下方按钮开始填写。电子签名：**

**[后附问卷内容]**

**问卷内容：**

一、单项选择题

1.您理解参与项目是自愿的，且可以随时撤回知情同意书并退出。您是否同意参与此项问卷调查，且满足参与的条件。

A．同意

B．不同意

2.您是否存在便秘的主观感受？

A．存在

B．不存在

3.您是否存在以下症状中的两项或以上：①在超过四分之一（25%）的排便过程中费力；②超过四分之一（25%）的排便中出现块状或硬便；③超过四分之一（25%）的排便感觉不尽；④超过四分之一（25%）的排便感觉肛门直肠梗阻/阻塞；⑤超过四分之一（25%）的手动操作以促进排便；⑥每周自发排便少于3次。

A．存在

B．不存在

4.不用泻药时是否很少出现稀粪？

A．是的，不用泻药时很少出现稀粪

B．不是，不用泻药时也常出现稀粪

5.以上症状出现至少6个月，这些症状必须至少持续三个月，并且在这段时间内症状一直存在？

A．是

B．否

6.工作或生活中是否站立、负重的时间多？

A．是

B．否

7.是否存在巨大儿（出生时体重大于4kg）分娩史？

A．是

B．否

8.是否存在慢性咳嗽、哮喘、慢性支气管炎？

A．是

B．否

1. 是否存在肠道恶性肿瘤、结直肠炎症病史？

A．是

B．否

1. 是否长期服用阿片类镇痛药或神经精神类药物

A．是

B．否

1. 是否存在子宫切除史

A．是

B．否

12.您的个人信息和问卷填写内容我们可能会将去识别化的数据用于未来科学研究：

1. 同意
2. 不同意
3. 填空题

1.您的姓名

2.您的年龄

3.您的中山大学附属第三医院登记号

4.您完成盆底超声检查的具体时间

5.您的身高 cm；体重 kg

Below is the English translation version.

**Survey Informed Consent Form**

**Research Title:** The Value of Pelvic Floor Ultrasound in the Assessment and Prediction of Chronic Constipation.

**Principal Investigator and Affiliation:** Bo Wei, Xinling Zhang, Department of Gastrointestinal Surgery, Department of Ultrasound, the Third Affiliated Hospital of Sun Yat-sen University

Dear Participant,

We are currently conducting a study that explores the pelvic floor structural characteristics in patients with constipation using ultrasound. This research will contribute to a deeper understanding of the pelvic floor anatomy involved in the onset of constipation and may help refine diagnostic and treatment guidelines for this condition. We invite you to participate in a questionnaire survey. Participation is entirely voluntary, and if you choose not to participate, your medical treatment and rights will not be affected. You may also change your mind and withdraw from the survey at any time. This project has been reviewed and approved by the Medical Ethics Committee.

**Eligibility Criteria for Participation in This Project**

- To participate in this questionnaire survey, you must meet the following criteria: Aged 40 years or older
- Have undergone pelvic floor ultrasound examination at the Third Affiliated Hospital of Sun Yat-sen University
- Possess the ability to understand the questionnaire and provide accurate answers

**Why Is This Project Being Conducted?**

The purpose of this project is to explore the impact of pelvic floor structural changes on constipation in female patients.

**How Will This Project Be Conducted?**

This questionnaire survey will ask questions about your constipation status and related risk factors. The questions will be in the form of multiple-choice and fill-in-the-blank. The survey will primarily be distributed via online platforms such as WeChat and Questionnaire Star, or by researchers in clinics and hospital wards. If you agree to participate, you will need to complete the questionnaire, and we will collect the responses for statistical analysis.

**Time/Cost Involved**

The questionnaire will take approximately 5 minutes to complete. Participating in this project will not incur any additional costs.

**Risk, Compensation, and Privacy Protection/Data Security**

Participating in this project will not affect your medical treatment and will not increase any risks.

In case of any harm related to this project, compensation will be provided according to the national laws and regulations if determined by the relevant authorities.

We will collect your personal information, including your name, age, height, and weight.

Your privacy will be protected, and the data you provide will be kept confidential. Your data will be stored on an encrypted hard drive and will be retained until the completion of the study. Only the research team for this project will have access to it. In necessary cases, government regulatory departments, research unit oversight bodies, or ethics review committees may access the data as per regulations.

The findings and results of this project may be used for academic publications, but your personally identifiable information will not be disclosed.

Your personal information and survey responses may be used in future scientific research in an anonymized format: □ I agree □ I do not agree

**Contact Information**

If you have any questions or need further information, please feel free to contact us.

Researcher Contact:

If you have any questions regarding your rights as a research participant, please contact: Ethics Committee of the Third Affiliated Hospital of Sun Yat-sen University.

**Consent Statement**

By agreeing, you confirm your participation in this questionnaire survey and that you meet the participation requirements.

You understand that participation in the project is voluntary, and you can withdraw your informed consent and exit the study at any time.

If you agree to the above statement, please click the button below to begin filling out the survey.

Electronic Signature:

[The questionnaire content is attached below]

**Questionnaire Content:**

**Section One: Multiple-Choice Questions**

1.You understand that participation in the project is voluntary, and you can withdraw your informed consent and exit the study at any time. Do you agree to participate in this questionnaire survey and meet the participation requirements?

A. Agree

B. Disagree

1.Do you have a subjective sensation of constipation?

A. Yes

B. No

1. Do you have two or more of the following symptoms: ① Straining during more than a quarter (25%) of your bowel movements; ② Hard lumps or hard stools in more than a quarter (25%) of your bowel movements; ③ Feeling of incomplete evacuation in more than a quarter (25%) of your bowel movements; ④ Feeling of anal or rectal obstruction or blockage in more than a quarter (25%) of your bowel movements; ⑤ Manual maneuvers to facilitate defecation in more than a quarter (25%) of your bowel movements; ⑥ Spontaneous bowel movements less than three times a week.

A. Yes

B. No

1. Do loose stools rarely occur without the use of laxatives?

A. Yes, loose stools rarely occur without the use of laxatives.

B. No, loose stools often occur even without the use of laxatives.

1. Have the above symptoms been present for at least 6 months, with the symptoms lasting at least 3 months, and consistently present during that time?

A. Yes

B. No

1. Do you spend a lot of time standing or carrying weight in your work or daily life?

A. Yes

B. No

1. Do you have a history of giving birth to a macrosomic infant (birth weight greater than 4 kg)?

A. Yes

B. No

1. Do you have a history of chronic cough, asthma, or chronic bronchitis?

A. Yes

B. No

1. Do you have a history of gastrointestinal malignancy or inflammatory bowel disease, such as colitis?

A. Yes

B. No

1. Do you take opioid painkillers or neuropsychiatric medications regularly?

A. Yes

B. No

11. Do you have a history of uterine removal (hysterectomy)?

A. Yes

B. No

1. We may use de-identified data from your personal information and questionnaire responses for future scientific research:

A. Agree

B. Disagree

**Section Two: Fill-in-the-Blank Questions**

1.Your name:

2.Your age:

3.Registration number at the Third Affiliated Hospital of Sun Yat-sen University:

4.The specific date of your pelvic floor ultrasound exam:

5.Your height: ____ cm; Weight: ____ kg.
